# Metabolic and cardiovascular risk in patients with a history of differentiated thyroid carcinoma: A case-controlled cohort study

**DOI:** 10.1186/1756-6614-1-2

**Published:** 2008-09-29

**Authors:** Massimo Giusti, Lorenzo Mortara, Roberta Degrandi, Francesca Cecoli, Michele Mussap, Guido Rodriguez, Diego Ferone, Francesco Minuto

**Affiliations:** 1 Clinica Endocrinologica, Azienda Ospedaliera Universitaria "San Martino", Genoa, Italy; 2 Laboratorio Analisi, Azienda Ospedaliera Universitaria "San Martino", Genoa, Italy; 3 Neurofisiologia Clinica, Azienda Ospedaliera Universitaria "San Martino", Genoa, Italy

## Abstract

Hyperthyroidism seems to increase metabolic and cardiovascular risk, while the effects of sub-clinical hyperthyroidism are controversial. We evaluated metabolic and cardiovascular parameters in differentiated thyroid carcinoma (DTC) patients with suppressed thyrotropin (TSH) due to levo-thyroxine (L-T4) therapy. We studied DTC patients and, as a control group, patients with a history of surgery for non-malignant thyroid pathology. Significantly higher insulin and lower HDL-cholesterol levels were recorded in DTC subjects. In both groups, insulin levels were significantly related with body mass index (BMI) but not with age or L-T4 dosage. In DTC patients, a significant negative correlation was seen between HDL-cholesterol and BMI or L-T4 dosage. In both groups, intima-media thickness (IMT) correlated positively with age, BMI, glucose levels and systolic blood pressure. In DTC patients, increased IMT was significantly correlated with glycated hemoglobin (HbA1c), cholesterol and triglycerides. In DTC patients, C-reactive protein correlated positively with insulin, insulin resistance, triglycerides and systolic blood pressure, and negatively with HDL-cholesterol. In both DTC and control subjects, fibrinogen correlated positively with age, BMI, increased IMT, HbA1c and systolic blood pressure. In DTC subjects, plasma fibrinogen concentrations correlated positively with insulin resistance, cholesterol and LDL-cholesterol, and negatively with TSH levels. Our data confirm that the favorable evolution of DTC can be impaired by a high incidence of abnormal metabolic and cardiovascular data that are, at least in part, related to L-T4 therapy. These findings underline the need for adequate L-T4 titration.

## Introduction

The incidence of thyroid cancer has increased markedly over the past few decades in several countries, reaching about 3% of newly diagnosed malignancies [1]. This increase can be ascribed to the widespread use of sonography techniques and of fine needle biopsy of thyroid nodules rather than to known environmental causes of thyroid cancers. In Italy, the surgical evaluation both of cold thyroid nodules [2] and of cytologically indeterminate or suspect nodules [3,4] indicates a 5–6% rate of malignancy. After total thyroidectomy and radioiodine ablation of thyroid remnants, all thyroid cancer patients require lifelong substitutive treatment to prevent hypothyroidism and thyrotropin (TSH)-related cell growth stimulation [5]. While thyroid hormone treatment improves disease-free survival, TSH-suppressive treatment must take into account the risk of causing iatrogenic damage due to prolonged exogenous hyperthyroxinemia [5].

Obesity and metabolic diseases are serious health problems in modern society. Indeed, body mass index (BMI), the most widely used indicator of obesity, has been seen to be positively related to a greater risk of mortality and morbidity [6], particularly cardiovascular diseases, diabetes mellitus, stroke and cerebrovascular diseases, osteoarticular diseases and, in some studies, malignancy [7]. Several studies have tried to evaluate the relationship between metabolic diseases and cancer; however, the data are conflicting [8-10].

A crucial factor in the outcome of cancer patients is comorbidity. In some patients, comorbidity may affect the presentation and recognition of symptoms, have prognostic significance, and increase the complexity of care. In a study conducted in newly diagnosed patients with differentiated thyroid cancer (DTC), one or more concomitant diseases were recorded in 32% [11]. The study showed that cardiovascular diseases and/or diabetes mellitus were present in 6% of DTC patients on diagnosis. Moreover, the finding that hypertension was the most frequent comorbidity (18%) followed by "other cancers" (7%), suggests that the concomitance of cardiovascular disease is probably underestimated [11].

Carotid artery intima-media thickness (IMT) is thought to predict coronary heart disease and stroke [12] and is an accepted marker of sub-clinical atherosclerosis [13]. Recently, Franzoni et al. [14] reported that patients with sub-clinical hypothyroidism showed an increase in carotid IMT. To our knowledge, no data on carotid IMT in patients with DTC have been reported.

Our aim was to evaluate several metabolic and cardiovascular parameters, including carotid artery IMT, in a cohort of patients with DTC. This evaluation was carried out in addition to the assessment of clinical and biochemical parameters indispensable for judging the evolution of DTC. We used a cohort of well-matched subjects with a history of partial or total surgery for non-malignant thyroid pathology as a control group. In both groups of subjects, metabolic parameters and parameters linked to cardiovascular risk were correlated with thyroid hormones, TSH and levo-thyroxine (L-T4) treatment. Our data confirm that DTC has a generally favorable evaluation. In addition, the present study shows a high incidence of abnormal metabolic and cardiovascular data, which are, at least in part, related to L-T4 therapy. Such comorbidity constitutes an additional risk factor over time. In particular, low HDL-cholesterol and high insulin levels, and a significant correlation between fibrinogen and TSH levels, underline the need for adequate L-T4 titration.

## Methods

### Subjects

The study was conducted on a cohort of 106 outpatients (mean age ± SD: 56.7 ± 13.5 years; range 21–85 years) with DTC diagnosed < 1 to 25 years earlier. Eighty-four were females (57.0 ± 13.6 years; range 21–82 years) and 22 were males (57.4 ± 13.7 years; range 28–85 years). Histology revealed papillary cancer, follicular variant of papillary cancer, medullary carcinoma, follicular cancer and insular cancer in 75, 16, 7, 5 and 3 subjects, respectively. Total thyroid ablation by near-total thyroidectomy and subsequent radioiodine therapy (for carcinomas of follicular origin) was our standard treatment. Only 16 patients with carcinomas of follicular origin did not undergo radioiodine therapy, for the following reasons: sub-total thyroidectomy (n = 3), no indication (microcarcinomas: n = 12), and refusal (n = 1). All subjects were on a TSH-suppressive L-T4 regimen at the time of examination. In 8 DTC subjects, TSH levels were in the normal (n = 4) or upper-normal (n = 4) range, owing to lack of compliance with the L-T4 regimen (n = 5) or recent L-T4 withdrawal for therapeutic purposes (n = 3). A cohort of 87 outpatients (56.5 ± 15.9 years; range 21–88 years) who had undergone thyroid surgery for benign thyroid diseases from < 1 to 51 years earlier, and under L-T4 regimen, served as a control group. Seventy-eight control subjects were females (56.1 ± 16.0 years, range 21–88 years) and 9 were males (60.0 ± 14.9 years; range 30–76 years). Subjects with known hypertension, diabetes or lipid abnormalities were included in the study. Participants were classified as non-smokers, former smokers or current smokers. In all patients, estimated alcohol intake was < 20 g/day. Written informed consent was obtained from all participants.

### Protocol

Clinical examination comprised pharmacological history, neck palpation, BMI (kg/m^2^) evaluation and blood pressure measurement after a 15 min rest in the supine position by means of a traditional (Riva-Rocci, Erka, Bad Tölz, Germany) mercury brachial cuff sphygmomanometer. Neck ultrasonography was performed with a 7.5 MHz linear probe (AU 5 Idea, Esaote, Genoa, Italy). Carotid artery IMT was evaluated on both sides by ultrasonography in the supine position. Color duplex ultrasound scanning was performed with Caris Plus (Esaote) and a 7.5 MHz linear-array transducer. The probe was used together with the resolution box function of the system, and setting were made in such a way as to produce an optimal picture of the carotid walls. Only the far walls of the artery were used for calculation. The IMT of the common carotid artery was defined as the distance between characteristics echoes from the lumen-intima and media adventitia interfaces. IMT was measured over a length of 1 cm just proximal to the bulb by 3 different measurements over this length; the mean value was then computed without including plaques. The IMT over both sides of carotid arteries was computed and designated as the mean IMT. Biochemical evaluation, which was performed in the fasting condition in the morning, comprised free-thyroid hormones, TSH, thyroglobulin (Tg), creatinine, glucose, insulin, glycated hemoglobin (HbA1c), total-cholesterol, high-density lipoprotein (HDL) cholesterol, triglycerides, C-reactive protein (CRP), and fibrinogen. Anti-Tg autoantibody (TgAb) was evaluated to identify sera in which a Tg-recovery test was necessary in order to obtain reliable Tg values. Calcitonin (CT) was assayed only in DTC of non-follicular origin. Homeostasis model assessment was used as a measure of insulin resistance (HOMA-IR), using the equation: fasting insulin (mU/l) × glucose (mmol/l)/22.5 [15].

### Assays

Serum Tg was assayed by chemiluminescence immunoassay (Roche Diagnostics, Mannheim, Germany). The functional sensitivity of the method is ≤ 0.5 μg/l. In our laboratory the intra- and inter-assay CVs were 5% and 8%. On the basis of the functional sensitivity of the methods, we selected 0.5 μg/l as the cut-off value to discriminate undetectable from detectable Tg levels. Tg-antibodies were measured by commercial assay (DiaSorin, Saluggia, Italy). A concentration of 100 mIU/l IgG to Tg was taken as the cut-off value. Sera in which TgAb was positive (> 100 mIU/l) were further processed by means of a Tg recovery test in order to determine the right Tg level. Serum CT assay was performed by chemiluminescence immunoassay (DiaSorin); the functional sensitivity of the method is ≤ 4.0 ng/l. In our laboratory the intra- and inter-assay CVs were 10% and 15%; in our laboratory the normal range of CT is < 10.0 ng/l. Free thyroid hormones and TSH were measured by means of ultra-sensitive chemiluminescence immunoassays (Roche Diagnostics). Normal ranges are 0.3–4.2 mIU/l for TSH, and 3.9–6.8 pmol/l and 12.0–22.0 pmol/l for free-T3 (f-T3) and free-T4 (f-T4), respectively. Serum creatinine (normal range: 44–115 μmol/l), glucose (3.3 – 5.8 mmol/l), cholesterol (3.4 – 5.2 mmol/l), HDL-cholesterol (> 1.6 mmol/l) and triglycerides (0.5 – 1.9 mmol/l) were determined by fully automated Modular P-800 Roche. Low-density lipoprotein (LDL)-cholesterol was calculated by means of the Friedewald equation [16] for those specimens with triglycerides < 2.7 mmol/l. In our laboratory, LDL-cholesterol values < 3.6 mmol/l are considered normal. HbA1c was measured by cation-exchange high-performance liquid chromatography with the TSKgel G7 Variant His column (Tosoh Co. Tokyo, Japan). Values are reported as percentages; in our laboratory, the normal range of HbA1c is 4.3–5.8%. Insulin was determined by means of a micro-particle enzyme immunoassay (Abbot, Abbot Park, IL, USA). In our laboratory, the normal range of insulin is 2.0–25.0 mU/l. Serum CRP and plasma fibrinogen were analyzed by commercial assays (Dade Behring, Marburg, Germany). Normal values in healthy subjects are < 3.0 mg/l for CRP and 180–350 g/l for fibrinogen.

### Statistical analysis

Data from DTC and control subjects were analyzed by means of the Prism 4.0 software (GraphPad Software, San Diego, CA, USA). To compare absolute and percentage data, the Mann-Whitney test and χ^2 ^test were used, as appropriate. Correlation analyses between variables were carried out by Spearman correlation test. All values quoted are means ± SEM. Data below the functional sensitivity of the assay were analyzed for statistical purposes by using the functional sensitivity value. Significance was taken as P < 0.05. For statistical purposes, the mean IMT was assigned a score ranging from 1 to 4 according to the following thickness: 1 = <0.6 mm, 2 = 0.6–0.7 mm, 3 = 0.8–0.9 mm, and 4 = >1.0 mm.

## Results

### Clinical data

Table 1 reports clinical data recorded in the two groups of subjects studied. The female-to-male ratio was 4:1 in DTC patients and 9:1 in controls. The weekly L-T4 dosage was significantly higher (P < 0.0001) in DTC than in control subjects. The two groups were well matched in terms of age, body weight, mean interval from diagnosis to study-period, incidence of patients with hypertension, diabetes mellitus or hyperlipidemia, history of cerebrovascular disease and smoking (Table 1). Both DTC patients (Spearman coefficient of correlation r_s _= 0.23, P = 0.02) and controls (r_s _= 0.47, P < 0.0001) showed a significant positive correlation between BMI and age. In DTC patients, the weekly L-T4 dosage showed a positive correlation with BMI (r_s _= 0.28, P = 0.004) and a negative correlation with age (r_s _= -0.22, P = 0.02), while a significant negative correlation was noted in controls only between weekly L-T4 dosage and age (r_s _= -0.40, P = 0.001). Except for one DTC patient with slight chronic renal insufficiency (creatinine 186 μmol/l), all subjects showed normal renal function (data not reported).

**Table 1:** Some demographic and clinical data observed in the whole group of DTC subjects and in controls.

	**DTC subjects**	**Control subjects**	**Significance**
Age (years)	57.0 ± 13.7	56.5 ± 15.9	ns
Sex (male-to-female ratio)	4 : 1	9 : 1	ns
Mean diagnosis-study interval (years)	7.0 ± 0.6	9.5 ± 1.0	ns
L-T4 dosage (μg/week)	863 ± 17	658 ± 26	P < 0.0001
BMI (kg/m^2^)	27.0 ± 0.5	25.6 ± 0.4	ns
Systolic blood pressure (mmHg)	128 ± 2	129 ± 2	ns
Diastolic blood pressure (mmHg)	82 ± 1	80 ± 1	ns
Hypertension	59 (56%)	46 (53%)	ns
Use of antihypertensive drugs	53 (50%)	39 (45%)	ns
Diabetes mellitus	5 (5%)	7 (8%)	ns
Use of antidiabetic drugs	4 (4%)	4 (5%)	ns
Use of dyslipidemia drugs	8 (7%)	6 (7%)	ns
Smokers	30 (28%)	35 (40%)	ns
Current smokers	13 (12%)	17 (19%)	-
Former smokers	17 (16%)	18 (21%)	-
History of myocardial infarction	0 (0%)	0 (0%)	ns
History of stroke	0 (0%)	0 (0%)	ns

Age is shown as mean ± SD, while the other continuous data are shown as mean ± SEM; nominal data are shown as absolute numbers and percentage values.

### Thyroid hormones, TSH, and tumor markers

Table 2 reports the average levels of f-T3, f-T4 and TSH in DTC and control subjects. As expected, owing to the higher weekly L-T4 dosage, DTC patients showed more elevated f-T4 concentrations (P < 0.0001) and more suppressed TSH levels (P < 0.0001) than controls (Table 2). On the basis of undetectable Tg values and negative neck sonography, 81% of subjects with DTC of follicular origin were considered disease-free. Active disease was found in 6% of patients with DTC of follicular origin, while in 13% Tg levels were detectable but less than 1 μg/L (n = 5). Baseline CT levels were elevated in 57% (n = 4) of DTC patients with medullary thyroid cancer.

**Table 2:** Serum levels of f-T3, f-T4, and TSH observed in the whole group of DTC subjects and in controls.

	**DTC subjects**	**Control subjects**	**Significance**
f-T3 (pmol/L)	4.4 ± 0.1	4.2 ± 0.1	P = 0.02
Median	4.5	4.1	
25^th ^percentile	3.8	3.8	
75^th ^percentile	5.1	4.7	
			
f-T4 (pmol/L)	20.1 ± 0.5	17.1. ± 0.5	P < 0.0001
Median	20.5	17.1	
25^th ^percentile	18.1	14.5	
75^th ^percentile	22.8	18.8	
			
TSH (mIU/L)	1.95 ± 0.86	2.94 ± 0.59	P < 0.0001
Median	0.16	1.30	
25^th ^percentile	0.05	0.75	
75^th ^percentile	0.60	2.47	

Data are reported as mean ± SEM and also as median, 25^th ^percentile and 75^th ^percentile.

### Metabolic parameters

Metabolic parameters are reported in table 3. No significant inter-group differences were noted in glucose, HbA1c or HOMA-IR levels. However, serum insulin levels were slightly but significantly (P = 0.05) higher in DTC patients than control subjects (Table 3). Among serum lipid parameters, the only significant (P = 0.01) difference was seen in the level of HDL-cholesterol in DTC patients (Table 3). Insulin levels correlated significantly, both in DTC (r_s _= 0.50, P < 0.0001) and control (r_s _= 0.37, P = 0.001) subjects, with BMI but not with age, L-T4 dosage or thyroid parameters, with the exception of TSH levels in controls (r_s _= 0.30, P = 0.01). Only in DTC patients was there a significant negative correlation between HDL-cholesterol and both BMI (r_s _= -0.42; P < 0.0001) and L-T4 dosage (r_s _= -0.29; P < 0.004), and a significant positive correlation between HDL-cholesterol and f-T4 levels (r_s _= 0.22, P = 0.03). The mean IMT score was similar in the DTC (2.6 ± 0.1) and control (2.5 ± 0.1) groups. In both groups, the IMT score correlated positively with age (DTC: r_s _= 0.68, P < 0.0001; controls: r_s _= 0.62, P < 0.0001), BMI (DTC: r_s _= 0.31, P = 0.002; controls: r_s _= 0.44, P < 0.0001), glucose levels (DTC: r_s _= 0.22, P = 0.03; controls: r_s _= 0.31, P = 0.008), systolic blood pressure (DTC: r_s _= 0.28, P = 0.005; controls: r_s _= 0.27, P = 0.02) and the number of hypotensive drugs administered (DTC: r_s _= 0.36, P = 0.0002; controls: r_s _= 0.31, P = 0.008), while it was unrelated to thyroid hormones, TSH, L-T4 dosages, insulin, HOMA-IR, HDL- and LDL-cholesterol, diastolic blood pressure or smoking. Only in the DTC patients did IMT significantly correlate with HbA1c (r_s _= 0.38; P = 0.0001), cholesterol (r_s _= 0.23, P = 0.02) and triglycerides (r_s _= 0.35, P = 0.0004).

**Table 3:** Several metabolic and inflammatory parameters observed in the whole group of DTC subjects and in controls.

	**DTC subjects**	**Control subjects**	**Significance**
Glucose (mmol/L)	4.7 ± 0.1	4.9 ± 0.1	ns
Insulin (mU/L)	8.5 ± 0.5	7.7 ± 0.6	P = 0.05
HOMA-IR [(mU × mmol)/22.5]	1.85 ± 0.13	1.74 ± 0.17	ns
HbA1c (%)	5.7 ± 0.1	5.7 ± 0.1	ns
Cholesterol (mmol/L)	5.36 ± 0.10	5.51 ± 0.11	ns
HDL-cholesterol (mmol/L)	1.71 ± 0.04	2.04 ± 0.09	P = 0.01
LDL-cholesterol (mmol/L)	3.11 ± 0.09	2.93 ± 0.11	ns
Triglycerides (mmol/L)	1.26 ± 0.06	1.21 ± 0.07	ns
CRP (mg/l)	4.4 ± 0.4	4.2 ± 0.3	ns
Fibrinogen (g/L)	355 ± 6	363 ± 10	ns

Data are reported as mean ± SEM.

### Inflammatory markers

**Figure 1:**
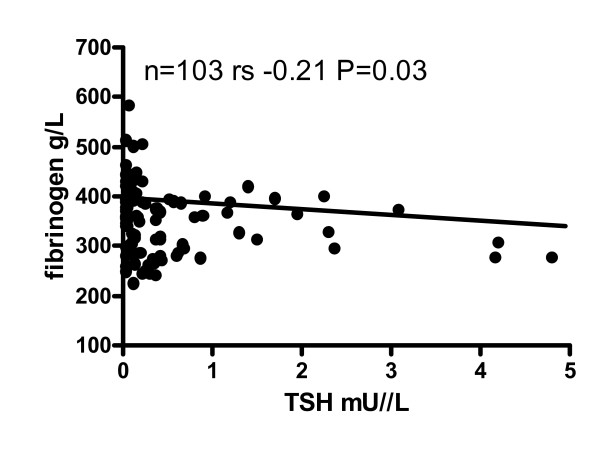
**Correlation between TSH and fibrinogen levels in the cohort of subjects with DTC.** While the number of pairs and significance for the analysis are reported, two experimental values of TSH above 5 mIU/L are not included in the graph.

CRP and fibrinogen levels, evaluated as inflammatory parameters, are reported in table 3. In both groups of subjects, mean CRP and fibrinogen levels were very similar and significantly (P < 0.0001) correlated with each other. Only in DTC patients did CRP positively correlate with insulin (r_s _= 0.32, P = 0.001), HOMA-IR (r_s _= 0.33, P = 0.001), triglycerides (r_s _= 0.26, P = 0.01) and systolic blood pressure (r_s _= 0.20, P = 0.05), and negatively with HDL-cholesterol (r_s _= -0.25, P = 0.01). Fibrinogen levels above the upper normal range (> 350 g/L) were recorded in a large number of subjects in both the DTC (48% of cases) and control (47% of cases) groups. In both DTC and control subjects, fibrinogen correlated positively with age (DTC: r_s _= 0.38, P < 0.0001; controls: r_s _= 0.44, P < 0.0001), BMI (DTC: r_s _= 0.22, P = 0.02; controls: r_s _= 0.41, P = 0.0002), IMT score (DTC: r_s _= 0.24, P = 0.02; controls: r_s _= 0.28 P = 0.02), HbA1c (DTC: r_s _= 0.39, P < 0.0001; controls: r_s _= 0.58, P < 0.0001) and systolic blood pressure (DTC: r_s _= 0.29, P = 0.004; controls r_s _= 0.24, P = 0.03). Only in DTC subjects did plasma fibrinogen concentrations correlate positively with HOMA-IR (r_s _= 0.21, P = 0.04), cholesterol (r_s _= 0.24, P = 0.02) and LDL-cholesterol (r_s _= 0.21, P = 0.03), and negatively (P = 0.03) with serum TSH levels (Figure 1). Only in controls did plasma fibrinogen levels correlate positively with glucose (r_s _= 0.29, P = 0.01) levels and the number of hypotensive drugs administered (r_s _= 0.40, P = 0.0004).

## Discussion

DTC patients have a good prognosis and survival [5]. While DTC can arise in young age, its incidence generally increases over time [1]. Our DTC subjects ranged from young to elderly, with a median age of 57 years at the moment of the present study and a median age of 53 years on diagnosis. Thyroid carcinomas are 3 times as frequent in females as in males [1]; a similar ratio was also seen in our study population. On the basis of undetectable Tg levels and negative neck sonography, in our study a disease-free condition was observed in 81% of patients with DTC of follicular origin, which is in line with the reported good prognosis of this cancer.

The medical therapy of follicular-origin DTC focuses on TSH suppression by means of L-T4 administration. In a meta-analysis involving 4174 patients (69% of cases with TSH < 0.1 mU/L), McGriff et al. [17] found a lower risk of recurrence, progression and disease-related death among patients on TSH-suppressive therapy. TSH suppression therefore remains the gold standard and only in low-risk tumors with favorable disease evolution is a reduction in L-T4 dosage countenanced. In our subjects, the L-T4 dosage was significantly higher in DTC patients than in controls, and TSH levels were correspondingly lower. Indeed, suppressed TSH levels (< 0.1 mIU/L) were found in 38% of DTC patients and only in 3% of controls. In the Colorado study, in which 6% of subjects were on L-T4 treatment, about 40% of these latter presented an abnormal TSH level [18]. In our study, too, both DTC and control subjects sometimes showed an abnormal TSH level (e.g. recent surgery, recent L-T4 withdrawal for radioiodine therapy, incorrect L-T4 administration, inadequate dosage, low compliance); we therefore feel that the efficacy of L-T4 therapy should be routinely checked to prevent under- or over-treatment. Exogenous hyperthyroxinemia accompanied by undetectable TSH levels and/or f-T4 above the therapeutic target has been found in 14% – 21% of patients under L-T4 [19,20]; in this situation, the objective of treatment should be weighed, on account of its well-known, potentially dangerous effects [21].

As cardiovascular and metabolic complications may be induced by L-T4 treatment, our goal was to evaluate DTC patients with regard to such complications, which could affect prognosis and life expectancy. Recently, in patients with thyroid cancer, concomitant diseases were found in 32%, hypertension being the most frequent [11]. In our study, the same prevalence of hypertension was noted in DTC and control subjects; hypertension seemed to be more closely linked to age, BMI and other independent cardiovascular risk factors, such as IMT and fibrinogen, than to L-T4 and thyroid hormones. Moreover, our data do not indicate a marked correlation between DTC and glucose control, in that the same percentage of subjects with impaired short- and medium-term glucose control and HOMA-IR levels was found among DTC and control subjects matched for BMI and age. Although higher insulin levels were found in DTC patients, the related exogenous hyperthyroxinemia did not induce the insulin resistance reported by some [22,23] but not all [24,25] authors with regard to overt endogenous hyperthyroxinemia. Moreover, the restoration of euthyroidism does not generally affect glucose metabolism in patients with DTC and long-term exogenous hyperthyroxinemia [24] and no correlation between current L-T4 dosage, TSH or free thyroid hormone levels and the evaluated parameters of glucose metabolism is reported [our data]. On the other hand, we observed a negative correlation between current L-T4 dosage and glucose levels, as well as a positive correlation between TSH and insulin levels in controls. This inter-group discrepancy could be due to the small number of subjects evaluated in our study and in similar studies [24,26]. By contrast, several literature data are available on the relationship between glucose metabolism and sub-clinical [27,28] or overt [26,29] hyperthyroidism, which are functional thyroid conditions that can be encountered during L-T4 titration even in subjects who have undergone thyroidectomy for malignant or non-malignant disease. Moreover, it cannot be excluded that fluctuations in thyroid hormones and TSH might, over time, affect glyco-metabolic parameters [30].

It is well known that lipid metabolism can be deranged both in sub-clinical and overt hypothyroidism [31,32]. While L-T4 treatment can improve dyslipidemia, the long-term effect of the L-T4 load on cardiovascular risk is unknown [33,34]. Overt hyperthyroidism is associated with reduced total- and HDL-cholesterol concentrations [35,36], which normalize after remission of hyperthyroidism [37]. Several studies have found no difference in lipid profiles in sub-clinical hyperthyroidism [38,39], though reduced cholesterol levels have sometimes been reported in subjects older than 55 years [40]. In our control and DTC subjects, cholesterol and triglyceride levels were not significantly different. However, HDL-cholesterol levels were significantly lower in DTC patients than in controls, and correlated negatively with L-T4 dosage and positively with f-T4. A similar reduction in HDL-cholesterol has been observed in sub-clinical hyperthyroidism and in L-T4 treated patients [31]. Recently, Regalbuto et al. [41] reported an increase in cholesterol levels in 45% of DTC patients before diagnostic L-T4 discontinuation. Other authors, however, have not found lipid profile changes in DTC patients before [24] and after [23,24] L-T4 dosage reduction. Moreover, in an old study, exogenous hyperthyroxinemia was reported to be associated with low total- and LDL-cholesterol [40]. In overt hyperthyroidism, triglyceride levels have been reported to be reduced [42], normal [43] or increased [44].

Non-invasive carotid artery IMT evaluation is an accepted index of cardiovascular risk and a pre-clinical marker of arteriosclerosis that is influenced by sex, age, smoking, lipid profile and hemodynamics [12,13]. As changes in lipid profile and hemodynamics may occur in thyroid diseases, interest in IMT evaluation has grown. In one cohort study, increased IMT correlated negatively with f-T4 levels in euthyroid subjects even after adjustment for age, sex and lipid profile [45]. However, IMT has been seen to be greater in both sub-clinical [14,46] and overt hypothyroidism [47], and can be reduced by L-T4 treatment [46,47]. In our study, no difference in IMT score or percentage of subjects with carotid stenosis was observed between the two cohorts of subjects. At present, it cannot be excluded that longitudinal studies or studies involving larger numbers of subjects might elucidate the direct effect of thyroid disease and treatment on IMT, over and above the effect exerted by age, BMI, glucose levels and hypertension. A linear relationship between thyroid function and IMT was found in a large cohort of at least 45-year-old subjects without known thyroid disorders, the highest IMT values being recorded in hyperthyroxinemia subjects and the lowest in subjects with elevated TSH levels [48]. In that study, thyroid function was identified as an independent risk factor for increased IMT [48]. The association between IMT and endogenous or exogenous thyroid hyperfunction is regarded as biologically plausible because there is a known link between hyperthyroidism and peripheral vasodilatation, which leads to a decrease in renal perfusion and activation of the renin-angiotensin system [49]. Angiotensin II stimulates vascular smooth muscle cell growth [50] and matrix synthesis [51]. Vascular hypertrophy is associated with increased vascular stiffness, a phenomenon reported in diffuse toxic goiter [52]. Thus, increased IMT of the carotid artery may merely reflect an adaptive response to shear and tensile stress linked to the increase in heart rate and systolic blood pressure generally found in hyperthyroxinemia [52]. Thus, in DTC, the permanent hyperthyroxinemia needed to maintain TSH suppression could increase the risk of both arrhythmias [21] and arteriosclerosis [53].

CRP levels are thought to be a widely fluctuating marker of inflammation, and moderate CRP elevation is linked to subsequent cardiovascular events [28,54]. In subjects with sub-clinical hypothyroidism, CRP has been found to be positively related to insulin and higher than in control subjects with normal thyroid function [28]. However, CRP levels are not normalized by L-T4 administration in sub-clinical hypothyroidism [54] and CRP is not related to TSH levels [27]. On the other hand, in a small group of 15 mostly disease-free DTC patients, Horne et al. [55] reported higher CRP levels under exogenous hyperthyroxinemia than after diagnostic L-T4 withdrawal. According to these data, it could be hypothesized that a mild inflammatory state is present in DTC patients on LT4 therapy. Our data did not show any difference between subjects on L-T4 after thyroidectomy for malignant or non-malignant thyroid disease. However, in DTC subjects, CRP seems to be modulated by BMI and HOMA-IR, and CRP and fibrinogen are significantly related in both groups of subjects [our data, [55]].

Fibrinogen is another well-known independent risk factor for cardiovascular diseases [56]. High fibrinogen levels are reported in sub-clinical [56] and overt [55,57] hyperthyroidism, as well as in sub-clinical [58] and overt [57] hypothyroidism. About 50% of both our DTC and control subjects displayed an increase in fibrinogen levels, which was also positively related to age, BMI, IMT score, HbA1c and systolic blood pressure. However, only in DTC subjects did plasma fibrinogen levels correlate positively with HOMA-IR and total- and LDL-cholesterol, and negatively with TSH levels but not with L-T4 dosages, as also reported in L-T4 treated hypothyroid patients [59]. Even in over-treated DTC patients Horne et al. [55] reported an increase in fibrinogen levels and several other coagulation factors. On the whole, these data seem to indicate that TSH-suppression in DTC patients could support an increased coagulation risk factor, which is probably independent of disease status and L-T4 dosage. Finally, another study has shown that high fibrinogen levels are linked in hyperthyroidism to an elevation of von Willebrand factor and to altered platelet plug formation as an direct index of endothelial dysfunction [60]. However, further data need to be considered, including the direct endothelial effect of TSH [61] and the role of cytokines such as osteoprotegerin [62,63] and interleukin-6 and tumor necrosis factor [61].

## Conclusion

In conclusion, our study aims to draw greater attention to metabolic and cardiovascular parameters in both DTC and control subjects, in whom exogenous hyperthyroxinemia could increase metabolic and cardiovascular risk. In DTC patients, life-long evaluation, as well as L-T4 therapy, is mandatory. Moreover, in medullary thyroid carcinoma and in low-risk disease-free patients suffering from DTC of follicular origin, exogenous hyperthyroxinemia must be avoided even though it does not seem to modify most hepatic, muscular, cardiac and neuropsychological parameters [41]. In DTC, slightly increased fT4 levels can, in the long term, induce an increase in insulin and a decrease in HDL-cholesterol levels, which are unfavorable developments. In addition, the significant inverse correlation between fibrinogen and (suppressed) TSH levels, associated to increases in cytokines [62], fibrinogen [60] and the endothelial release of von Willebrand factor [55], seems to document a greater cardiovascular risk in DTC patients.

## Competing interests

The authors declare that they have no competing interests.

## Authors' contributions

MG conceive the study, performed the statistical analysis and drafted the manuscript. LM participated in the design of the study. RD carried out the assays. FC participated in the design of the study. MM participated in the coordination of the laboratory data. GR carried out the instrumental evaluations. DF participated in the sequence alignment. FM participated in the conceive of the study. All authors read and approved the final manuscript.

## References

[B1] Gorges R, Biersack H-J, Grunwald F (2005). The changing epidemiology of the thyroid cancer. Thyroid cancer.

[B2] Belfiore A, La Rosa GL, La Porta GA, Giuffrida D, Milazzo G, Lupo L, Regalbuto C, Vigneri R (1992). Cancer risk in patients with cold thyroid nodules: relevance of thyroid intake, sex, age and multinodularity. Am J Med.

[B3] Papini E, Guglielmi R, Bianchini A, Crescenzi A, Taccogna S, Nardi F, Panunzi C, Rinaldi R, Toscano V, Pacella CM (2002). Risk of malignancy in non-palpable thyroid nodules: predictive value of ultrasound and color Doppler features. J Clin Endocrinol Metab.

[B4] Sidoti M, Marino G, Resmini E, Augeri C, Cappi C, Cavallero D, Lagasio C, Ceppa P, Minuto F, Giusti M (2006). The rational use of fine needle aspiration biopsy (FNAB) in diagnosing thyroid nodules. Minerva Endocrinol.

[B5] Tuttle RM, Leboeuf R, Martorella AJ (2007). Papillary thyroid cancer and therapy. Endocrinol Metab Clin N Am.

[B6] Adams KF, Schatzkin A, Harris TB, Kipnis V, Mouw T, Ballard-Barbash R, Hollenbeck A, Leitzmann MF (2006). Overweight obesity and mortality in a large prospective cohort of persons 50 to 71 years old. N Engl J Med.

[B7] Conway B, Rene A (2004). Obesity as a disease: no light-weight matter. Obes Rev.

[B8] Adami HO, McLaughlin J, Ekbom A, Ekbom A, Berne C, Silverman D, Hacker D, Persson I (1991). Cancer risk in patients with diabetes mellitus. Cancer Causes Control.

[B9] Wideroff L, Gridley G, Mellemkjaer L, Chow WH, Linet M, Keehn S, Borch-Johnsen K, Olsen JH (1997). Cancer incidence in a population based cohort of patients hospitalized with diabetes mellitus in Denmark. J Natl Cancer Inst.

[B10] Rapp K, Schroeder J, Klenk J, Ulmer H, Concin H, Diem G, Oberaigner W, Weiland SK (2006). Fasting blood glucose and cancer risk in a cohort of more than 140,000 adults in Austria. Diabetologia.

[B11] Kuijpens JL, Janssen-Heijnen ML, Lemmens VE, Haak HR, Heijckmann AC, Coebergh JW (2006). Comorbidity in newly diagnosed thyroid cancer patients: a population-based study on prevalence and the impact on treatment and survival. Clin Endocrinol (Oxf).

[B12] Gnasso A, Irace C, Mattioli PL, Pujia A (1996). Carotid intima-media thickness and coronary heart disease risk factors. Atherosclerosis.

[B13] de Groot E, Hovingh GK, Wiegman A, Duriez P, Smit AJ, Fruchart JC, Kastelein JJ (2004). Measurement of arterial wall thickness as a surrogate marker for atherosclerosis. Circulation.

[B14] Franzoni F, Galetta F, Fallahi P, Tocchini L, Braccini L, Rossi M, Carpi A, Santoro G, Antonelli A (2008). Carotid integrated backscatter analysis in patients with subclinical hypothyroidism. Clin Endocrinol (Oxf).

[B15] Matthews DR, Hosker JP, Rudenski AS, Naylor BA, Treacher DF, Turner RC (1985). Homeostasis model assessment: insulin resistance and beta-cell function from fasting plasma glucose and insulin concentrations in man. Diabetologia.

[B16] Friedewald WT, Levy RI, Fredrickson DS (1972). Estimation of the concentration of low-density lipoprotein cholesterol in plasma, without use of the preparative ultracentrifuge. Clin Chem.

[B17] McGriff NJ, Csako G, Gourgiotis L, Lori CG, Pucino F, Sarlis NJ (2002). Effects of thyroid hormone suppression therapy on adverse clinical outcomes in thyroid cancer. Ann Med.

[B18] Canaris GJ, Manowitz NR, Mayor G, Ridgway EC (2000). The Colorado thyroid disease prevalence study. Arch Intern Med.

[B19] Parle JV, Franklyn JA, Cross KW, Jones SR, Sheppard MC (1993). Thyroxine prescription in the community: serum thyroid stimulating hormone level assays as an indicator of undertreatment or overtreatment. Br J Gen Pract.

[B20] Ross DS, Daniels GH, Gouvela D (1990). The use and limitations of chemiluminescent thyrotropin assay as a single thyroid function test in out-patient endocrine clinic. J Clin Endocrinol Metab.

[B21] Surks MI, Ortiz E, Daniels GH, Sawin CT, Col NF, Cobin RH, Franklin JA, Hershman JM, Burman KD, Denke MA, Gorman C, Cooper RS, Weissman NJ (2004). Subclinical thyroid disease: scientific review and guidelines for diagnosis and management. JAMA.

[B22] Kreines K, Jett M, Knowles HC (1965). Observations in hyperthyroidism of abnormal glucose tolerance and other traits related to diabetes mellitus. Diabetes.

[B23] Gimenez-Palop O, Gimenez-Perez G, Mauricio D, Berlanga E, Potau N, Vilardell C, Arroyo J, Gonzalez-Clemente JM, Caixas A (2005). Circulating ghrelin in thyroid dysfunction is related to insulin resistance and not to hunger, food intake or anthropometric changes. Eur J Endocrinol.

[B24] Heemstra KA, Smit JW, Eustatia-Rutten CF, Heijboer AC, Frolich M, Romijn JA, Corssmit EP (2006). Glucose tolerance and lipid profile in longterm exogenous subclinical hyperthyroidism and the effects of restoration of euthyroidism, a randomised controlled trial. Clin Endocrinol (Oxf).

[B25] Owecki M, Nikisch E, Sowinski J (2006). Hypothyroidism has no impact on insulin sensitivity assessed with HOMA-IR in totally thyroidectomized patients. Acta Clin Belg.

[B26] Yavuz DG, Yukselt M, Deyneli O, Ozen Y, Aydin H, Akalin S (2004). Association of serum paraoxonase activity with insulin sensitivity and oxidative stress in hyperthyroid and TSH-suppressed nodular goitre patients. Clin Endocrinol (Oxf).

[B27] Al Sayed A, Al Ali N, Bo Abbas Y, Alfadhli E (2006). Subclinical hypothyroidism is associated with early insulin resistance in Kuwaiti women. Endocr J.

[B28] Tuzcu A, Bahceci M, Gokalp D, Tuzun Y, Gunes K (2005). Subclinical hypothyroidism may be associated with elevated high-sensitive c-reactive protein (low grade inflammation) and fasting hyperinsulinemia. Endocr J.

[B29] Takashima N, Niwa Y, Mannami T, Tomoike H, Iwai N (2007). Characterization of subclinical thyroid dysfunction from cardiovascular and metabolic viewpoints: the Suita study. Circ J.

[B30] Fernandez-Real JM, Lopez-Bermejo A, Castro A, Casamitijana R, Ricart W (2006). Thyroid function is intrinsically linked to insulin resistance and endothelium-dependent vasodilatation in healthy euthyroid subjects. J Clin Endocrinol Metab.

[B31] Duntas LH (2002). Thyroid disease and lipids. Thyroid.

[B32] Milionis HJ, Tambaki AP, Kanioglou CN, Elisaf MS, Tselepis AD, Tsatsoulis A (2005). Thyroid substitution therapy induces high density lipoprotein-associated platelet-activating factor-acetylidrolase in patients with subclinical hypothyroidism: a potential antiatherogenic effect. Thyroid.

[B33] Chubb S, Davis WA, Inman Z, Davis TM (2005). Prevalence and progression of subclinical hypothyroidism in women with type 2 diabetes: the Fremantle diabetes study. Clin Endocrinol (Oxf).

[B34] Danese MD, Landeson PW, Meinert CL, Powe NR (2000). Effect of thyroxine therapy on serum lipoproteins in patients with mild thyroid failure: a quantitative review of the literature. J Clin Endocrinol Metab.

[B35] Cachefo A, Boucher P, Vidon C, Dussere E, Diraison F, Beylot M (2001). Hepatic lipogenesis and cholesterol synthesis in hyperthyroid patients. J Clin Endocrinol Metab.

[B36] Iglesias P, Alvarez Fidalgo P, Codoceo R, Diez JJ (2003). Serum concentrations of adipocytokines in patients with hyperthyroidism and hypothyroidism before and after control of thyroid function. Clin Endocrinol (Oxf).

[B37] Oge A, Sozmen E, Karaoglu AO (2004). Effect of thyroid function on LDL oxidation in hypothyroidism and hyperthyroidism. Endocr Res.

[B38] Kung AW, Pang RW, Lauder I, Lam KS, Janus ED (1995). Changes in serum lipoprotein and lipids during treatment of hyperthyroidism. Clin Chem.

[B39] Parle JV, Franklyn JA, Cross KW, Jones SR, Sheppard MC (1992). Circulating lipids and minor abnormalities of thyroid function. Clin Endocrinol (Oxf).

[B40] Franklyn JA, Daykin J, Betteridge J, Haughes EA, Jones SR, Sheppard MC (1993). Thyroxin replacement therapy and circulating lipid concentration. Clin Endocrinol.

[B41] Regalbuto C, Alagona C, Maiorana R, Di Paola R, Cianci M, Alagona G, Sapienza S, Vigneri R, Pezzino V (2006). Acute changes in clinical parameters and thyroid function peripheral markers following L-T4 withdrawal in patients totally thyroidectomized for thyroid cancer. J Endocrinol Invest.

[B42] Raiszadeh F, Solati M, Etemandi A, Azizi F (2004). Serum paraoxonase activity before and after treatment of thyrotoxicosis. Clin Endocrinol (Oxf).

[B43] Lam KS, Chan MK, Yeung RT (1986). High density lipoprotein cholesterol, hepatic lipase and lipoprotein lipase activities in thyroid dysfunction – effects treatment. Q J Med.

[B44] Riis AL, Hansen TK, Moller N, Weeke J, Jorgensen JO (2003). Hyperthyroidism is associated with suppressed and circulating ghrelin levels. J Clin Endocrinol Metab.

[B45] Dullaart RP, de Vries R, Roozendaalt C, Koboldt AC, Sluiter WJ (2007). Carotid artery intima media thickness is inversely related to serum free thyroxine in euthyiroid subjects. Clin Endocrinol (Oxf).

[B46] Monzani F, Caraccio N, Kozakowa M, Dardano A, Vittobe F, Virdis A, Taddei S, Palombo C, Ferrannini E (2004). Effect of levothyroxine replacement on lipid profile and intima-media thickness in subclinical hypothyroidism: a double-blind placebo controlled study. J Clin Endocrinol Metab.

[B47] Nagasaki T, Inaba M, Henmi Y, Kumeda Y, Ueda M, Tahara H, Ishimura E, Onoda N, Ishikawa T, Nishizawa Y (2004). Change in von Willebrand factor and carotid intima-media thickness in hypothyroid patients with normal thyroid function after levothyroxine replacement therapy. Eur J Endocrinol.

[B48] Volzke H, Robinson DM, Schiminke U, Ludemann J, Rettig R, Felix SB, Kessler C, John U, Meng W (2004). Thyroid function and carotid wall thickness. J Clin Endocrinol Metab.

[B49] Garcia-Estan J, Atucha NM, Quesada T, Vargas F (1995). Involvement of the renin-angiotensin system in the reduced pressure natriuresis response of hyperthyroid rats. Am J Physiol.

[B50] Paul M, Ganten D (1992). The molecular basis of cardiovascular hypertrophy. The role of the rennin-angiotensin system. J Cardiovasc Pharmacol.

[B51] Kato H, Suzuki H, Tajima S, Ogata Y, Tominaga T, Sato A, Saruta T (1991). Angiotensin II stimulates collagen synthesis in cultured vascular smooth muscle cells. J Hypertens.

[B52] Czarkowski M, Hilgertner L, Powalowski T, Radomski D (2002). The stiffness of the common carotid artery in patients with Graves' disease. Int Angiol.

[B53] Volzke H, Schwahn C, Wallanschofski H, Dorr M (2007). The association of thyroid dysfunction with all-cause and circulatory mortality: is there a causal relationship?. J Clin Endocrinol Metab.

[B54] Kohler HP, Grant PJ (2000). Plasminogen-activator inhibitor type 1 and coronary artery disease. N Engl J Med.

[B55] Horne MK, Singh KK, Rosenfeld KG, Wesley R, Skarulis MC, Merryman PK, Cullinane A, Costello R, Patterson A, Egerman T, Bernstein DM, Pucino F, Csako G (2004). Is thyroid hormone suppression therapy prothrombotic?. J Clin Endocrinol Metab.

[B56] Dorr M, Robinson DM, Wallaschofski H, Schwahn C, John U, Felix SB, Volzke H (2006). Low serum thyrotropin is associated with high plasma fibrinogen. J Clin Endocrinol Metab.

[B57] Chaderevian R, Bruckert E, Giral P, Turpin G (1999). Relationship between thyroid hormones and fibrinogen levels. Blood Coagul Fibrinolysis.

[B58] Cakal B, Cakal E, Dernirbas B, Ozkaya M, Karaahmetoglu S, Serter R, Aral Y (2007). Homocysteine and fibrinogen changes with L-thyroxine in subclinical hypothyroid patients. J Korean Med Sci.

[B59] Canturk Z, Cetinarslan B, Tarkun J, Canturk NZ, Ozden M, Duman C (2003). Hemostatic system as a risk factor for cardiovascular disease in women with subclinical hypothyroidism. Thyroid.

[B60] Homoncik M, Gessl A, Ferlitsch A, Jilma B, Vierhapper H (2007). Altered platelet plug formation in hyperthyroidism and hypothyroidism. J Clin Endocrinol Metab.

[B61] Dardano A, Ghiadoni L, Plantinga Y, Caraccio N, Berni A, Duranti E, Taddei S, Ferrannini E, Salvetti A, Monzani F (2006). Recombinant human thyrotropin reduces endothelium dependent vasodilation in patient monitored for differentiated thyroid carcinoma. J Clin Endocrinol Metab.

[B62] Giusti M, Cecoli F, Fazzuoli L, De Franchis V, Ceresola E, Ferone D, Mussap M, Minuto F (2007). Serum osteoprotegerin and soluble receptor activator of nuclear factor kB ligand levels in patients with a history of differentiated thyroid carcinoma: a case-controlled cohort study. Metabolism.

[B63] Mikosch P, Igerc I, Kudlacek S, Woloszczuk W, Gallowitsch HJ, Krsnik E, Stettner H, Grimm G, Lind P, Pietschmann P (2006). Receptor activator nuclear factor kb ligand and osteoprotegerin in men with thyroid cancer. Eur J Clin Invest.

